# Dating Apps and Their Sociodemographic and Psychosocial Correlates: A Systematic Review

**DOI:** 10.3390/ijerph17186500

**Published:** 2020-09-07

**Authors:** Ángel Castro, Juan Ramón Barrada

**Affiliations:** Facultad de Ciencias Sociales y Humanas, Universidad de Zaragoza, Calle Atarazanas, 4. 44003 Teruel, Spain; barrada@unizar.es

**Keywords:** dating apps, Tinder, Grindr, systematic review

## Abstract

The emergence and popularization of dating apps have changed the way people meet and interact with potential romantic and sexual partners. In parallel with the increased use of these applications, a remarkable scientific literature has developed. However, due to the recency of the phenomenon, some gaps in the existing research can be expected. Therefore, the objective of this study was to conduct a systematic review of the empirical research of the psychosocial content published in the last five years (2016–2020) on dating apps. A search was conducted in different databases, and we identified 502 articles in our initial search. After screening titles and abstracts and examining articles in detail, 70 studies were included in the review. The most relevant data (author/s and year, sample size and characteristics, methodology) and their findings were extracted from each study and grouped into four blocks: user dating apps characteristics, usage characteristics, motives for use, and benefits and risks of use. The limitations of the literature consulted are discussed, as well as the practical implications of the results obtained, highlighting the relevance of dating apps, which have become a tool widely used by millions of people around the world.

## 1. Introduction

In the last decade, the popularization of the Internet and the use of the smartphone and the emergence of real-time location-based dating apps (e.g., Tinder, Grindr) have transformed traditional pathways of socialization and promoted new ways of meeting and relating to potential romantic and/or sexual partners [1,2,3,4].

It is difficult to know reliably how many users currently make use of dating apps, due to the secrecy of the developer companies. However, thanks to the information provided by different reports and studies, the magnitude of the phenomenon can be seen online. For example, the *Statista Market Forecast* [5] portal estimated that by the end of 2019, there were more than 200 million active users of dating apps worldwide. It has been noted that more than ten million people use Tinder daily, which has been downloaded more than a hundred million times worldwide [6,7]. In addition, studies conducted in different geographical and cultural contexts have shown that around 40% of single adults are looking for an online partner [8], or that around 25% of new couples met through this means [9].

Some theoretical reviews related to users and uses of dating apps have been published, although they have focused on specific groups, such as men who have sex with men (MSM [10,11]) or on certain risks, such as aggression and abuse through apps [12].

Anzani et al. [1] conducted a review of the literature on the use of apps to find a sexual partner, in which they focused on users’ sociodemographic characteristics, usage patterns, and the transition from online to offline contact. However, this is not a systematic review of the results of studies published up to that point and it leaves out some relevant aspects that have received considerable research attention, such as the reasons for use of dating apps, or their associated advantages and risks.

Thus, we find a recent and changing object of study, which has achieved great social relevance in recent years and whose impact on research has not been adequately studied and evaluated so far. Therefore, the objective of this study was to conduct a systematic review of the empirical research of psychosocial content published in the last five years (2016–2020) on dating apps. By doing so, we intend to assess the state of the literature in terms of several relevant aspects (i.e., users’ profile, uses and motives for use, advantages, and associated risks), pointing out some limitations and posing possible future lines of research. Practical implications will be highlighted.

## 2. Materials and Methods

The systematic literature review was conducted according to the Preferred Reporting Items for Systematic Reviews and Meta-Analyses (PRISMA) guidelines [13,14], and following the recommendations of Gough et al. [15]. However, it should be noted that, as the objective of this study was to provide a state of the art view of the published literature on dating apps in the last five years and without statistical data processing, there are several principles included in the PRISMA that could not be met (e.g., summary measures, planned methods of analysis, additional analysis, risk of bias within studies). However, following the advice of the developers of these guidelines concerning the specific nature of systematic reviews, the procedure followed has been described in a clear, precise, and replicable manner [13].

### 2.1. Literature Search and Inclusion/Exclusion Criteria

We examined the databases of the Web of Science, Scopus, and Medline, as well as PsycInfo and Psycarticle and Google Scholar, between 1 March and 6 April 2020. In all the databases consulted, we limited the search to documents from the last five years (2016–2020) and used general search terms, such as “dating apps” and “online dating” (linking the latter with “apps”), in addition to the names of some of the most popular and frequently used dating apps worldwide, such as “tinder”, “grindr”, and “momo”, to identify articles that met the inclusion criteria (see below).

The selection criteria in this systematic review were established and agreed on by the two authors of this study. The database search was carried out by one researcher. In case of doubt about whether or not a study should be included in the review, consultation occurred and the decision was agreed upon by the two researchers.

**Figure 1:**
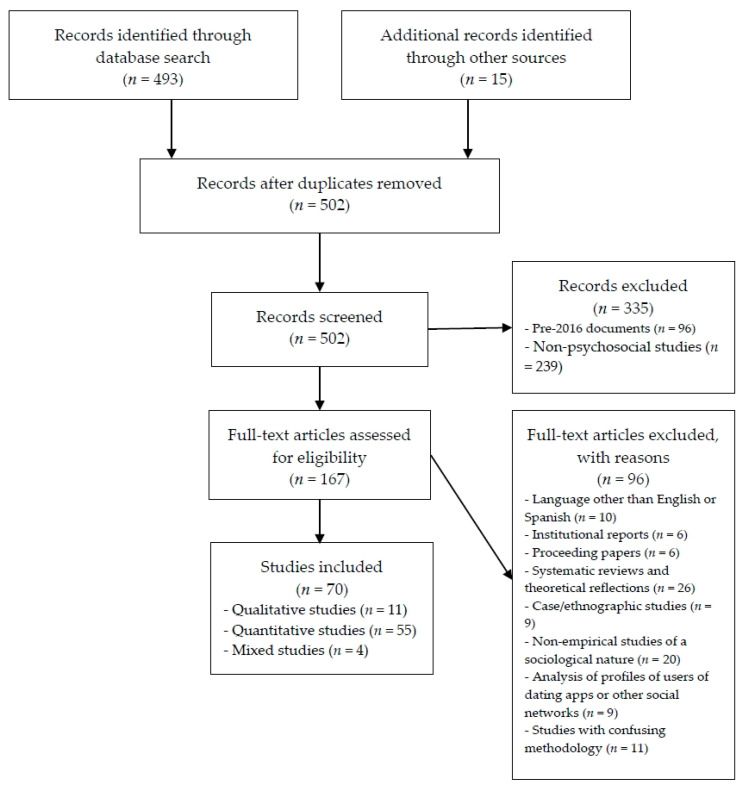
Flowchart of the systematic review process.

Four-hundred and ninety-three results were located, to which were added 15 documents that were found through other resources (e.g., social networks, e-mail alerts, newspapers, the web). After these documents were reviewed and the duplicates removed, a total of 502 records remained, as shown by the flowchart presented in Figure 1. At that time, the following inclusion criteria were applied: (1) empirical, quantitative or qualitative articles; (2) published on paper or in electronic format (including “online first”) between 2016 and 2020 (we decided to include articles published since 2016 after finding that the previous empirical literature in databases on dating apps from a psychosocial point of view was not very large; in fact, the earliest studies of Tinder included in Scopus dated back to 2016; (3) to be written in English or Spanish; and (4) with psychosocial content. No theoretical reviews, case studies/ethnography, user profile content analyses, institutional reports, conference presentations, proceeding papers, etc., were taken into account.

Thus, the process of refining the results, which can be viewed graphically in Figure 1, was as follows. Of the initial 502 results, the following exclusion criteria were applied: (1) pre-2016 documents (96 records excluded); (2) documents that either did not refer to dating apps or did so from a technological approach (identified through title and abstract; 239 records excluded); (3) published in a language other than English or Spanish (10 records excluded); (4) institutional reports, or analysis of the results of such reports (six records excluded); (5) proceeding papers (six records excluded); (6) systematic reviews and theoretical reflections (26 records excluded); (7) case studies/ethnography (nine records excluded); (8) non-empirical studies of a sociological nature (20 records excluded); (9) analysis of user profile content and campaigns on dating apps and other social networks (e.g., Instagram; nine records excluded); and (10) studies with confusing methodology, which did not explain the methodology followed, the instruments used, and/or the characteristics of the participants (11 records excluded). This process led to a final sample of 70 empirical studies (55 quantitative studies, 11 qualitative studies, and 4 mixed studies), as shown by the flowchart presented in Figure 1.

### 2.2. Data Collection Process and Data Items

One review author extracted the data from the included studies, and the second author checked the extracted data. Information was extracted from each included study of: (1) author/s and year; (2) sample size and characteristics; (3) methodology used; (4) main findings.

## 3. Results

Table 1 shows the information extracted from each of the articles included in this systematic review. The main findings drawn from these studies are also presented below, distributed in different sections.

### 3.1. Characteristics of Reviewed Studies

First, the characteristics of the 70 articles included in the systematic review were analyzed. An annual increase in production can be seen, with 2019 being the most productive year, with 31.4% (*n* = 22) of included articles. More articles (11) were published in the first three months of 2020 than in 2016. It is curious to note, on the other hand, how, in the titles of the articles, some similar formulas were repeated, even the same articles (e.g., Love me Tinder), playing with the swipe characteristic of this type of application (e.g., Swiping more, Swiping right, Swiping me).

As for the methodology used, the first aspect to note is that all the localized studies were cross-sectional and there were no longitudinal ones. As mentioned above, 80% (*n* = 55) of the studies were quantitative, especially through online survey (*n* = 49; 70%). 15.7% (*n* = 11) used a qualitative methodology, either through semi-structured interviews or focus groups. And 5.7% (*n* = 4) used a mixed methodology, both through surveys and interviews. It is worth noting the increasing use of tools such as Amazon Mechanical Turk (*n* = 9, 12.9%) or Qualtrics (*n* = 8, 11.4%) for the selection of participants and data collection.

The studies included in the review were conducted in different geographical and cultural contexts. More than one in five investigations was conducted in the United States (22.8%, *n* = 16), to which the two studies carried out in Canada can be added. Concerning other contexts, 20% (*n* = 14) of the included studies was carried out in different European countries (e.g., Belgium, The Netherlands, UK, Spain), whereas 15.7% (*n* = 11) was carried out in China, and 8.6% (*n* = 6) in other countries (e.g., Thailand, Australia). However, 21.4% (*n* = 15) of the investigations did not specify the context they were studying.

Finally, 57.1% (*n* = 40) of the studies included in the systematic review asked about dating apps use, without specifying which one. The results of these studies showed that Tinder was the most used dating app among heterosexual people and Grindr among sexual minorities. Furthermore, 35% (*n* = 25) of the studies included in the review focused on the use of Tinder, while 5.7% (*n* = 4) focused on Grindr.

### 3.2. Characteristics of Dating App Users

It is difficult to find studies that offer an overall user profile of dating apps, as many of them have focused on specific populations or groups. However, based on the information collected in the studies included in this review, some features of the users of these applications may be highlighted.

Gender. Traditionally, it has been claimed that men use dating apps more than women and that they engage in more casual sex relationships through apps [3]. In fact, some authors, such as Weiser et al. [75], collected data that indicated that 60% of the users of these applications were male and 40% were female. Some current studies endorse that being male predicts the use of dating apps [23], but research has also been published in recent years that has shown no differences in the proportion of male and female users [59,68].

To explain these similar prevalence rates, some authors, such as Chan [27], have proposed a feminist perspective, stating that women use dating apps to gain greater control over their relationships and sexuality, thus countering structural gender inequality. On the other hand, other authors have referred to the perpetuation of traditional masculinity and femmephobic language in these applications [28,53].

Age. Specific studies have been conducted on people of different ages: adolescents [49], young people (e.g., [21,23,71]), and middle-aged and older people [58]. The most studied group has been young people between 18 and 30 years old, mainly university students, and some authors have concluded that the age subgroup with a higher prevalence of use of dating apps is between 24 and 30 years of age [44,59].

Sexual orientation. This is a fundamental variable in research on dating apps. In recent years, especially after the success of Tinder, the use of these applications by heterosexuals, both men and women, has increased, which has affected the increase of research on this group [3,59]. However, the most studied group with the highest prevalence rates of dating apps use is that of men from sexual minorities [18,40]. There is considerable literature on this collective, both among adolescents [49], young people [18], and older people [58], in different geographical contexts and both in urban and rural areas [24,36,43,79]. Moreover, being a member of a sexual minority, especially among men, seems to be a good predictor of the use of dating apps [23].

For these people, being able to communicate online can be particularly valuable, especially for those who may have trouble expressing their sexual orientation and/or finding a partner [3,80]. There is much less research on non-heterosexual women and this focuses precisely on their need to reaffirm their own identity and discourse, against the traditional values of hetero-patriate societies [35,69].

Relationship status. It has traditionally been argued that the prevalence of the use of dating apps was much higher among singles than among those with a partner [72]. This remains the case, as some studies have shown that being single was the most powerful sociodemographic predictor of using these applications [23]. However, several investigations have concluded that there is a remarkable percentage of users, between 10 and 29%, who have a partner [4,17,72]. From what has been studied, usually aimed at evaluating infidelity [17,75], the reasons for using Tinder are very different depending on the relational state, and the users of this app who had a partner had had more sexual and romantic partners than the singles who used it [72].

Other sociodemographic variables. Some studies, such as the one of Shapiro et al. [64], have found a direct relationship between the level of education and the use of dating apps. However, most studies that contemplated this variable have focused on university students (see, for example [21,23,31,38]), so there may be a bias in the interpretation of their results. The findings of Shapiro et al. [64] presented a paradox: while they found a direct link between Tinder use and educational level, they also found that those who did not use any app achieved better grades. Another striking result about the educational level is that of the study of Neyt et al. [9] about their users’ characteristics and those that are sought in potential partners through the apps. These authors found a heterogeneous effect of educational level by gender: whereas women preferred a potential male partner with a high educational level, this hypothesis was not refuted in men, who preferred female partners with lower educational levels.

Other variables evaluated in the literature on dating apps are place of residence or income level. As for the former, app users tend to live in urban contexts, so studies are usually performed in large cities (e.g., [11,28,45]), although it is true that in recent years studies are beginning to be seen in rural contexts to know the reality of the people who live there [43]. It has also been shown that dating app users have a higher income level than non-users, although this can be understood as a feature associated with young people with high educational levels. However, it seems that the use of these applications is present in all social layers, as it has been documented even among homeless youth in the United States [66].

Personality and other psychosocial variables. The literature that relates the use of dating apps to different psychosocial variables is increasingly extensive and diverse. The most evaluated variable concerning the use of these applications is self-esteem, although the results are inconclusive. It seems established that self-esteem is the most important psychological predictor of using dating apps [6,8,59]. But some authors, such as Orosz et al. [55], warn that the meaning of that relationship is unclear: apps can function both as a resource for and a booster of self-esteem (e.g., having a lot of matches) or to decrease it (e.g., lack of matches, ignorance of usage patterns).

The relationship between dating app use and attachment has also been studied. Chin et al. [29] concluded that people with a more anxious attachment orientation and those with a less avoidant orientation were more likely to use these apps.

Sociosexuality is another important variable concerning the use of dating apps. It has been found that users of these applications tended to have a less restrictive sociosexuality, especially those who used them to have casual sex [6,7,8,21].

Finally, the most studied approach in this field is the one that relates the use of dating apps with certain personality traits, both from the Big Five and from the dark personality model. As for the Big Five model, Castro et al. [23] found that the only trait that allowed the prediction of the current use of these applications was open-mindedness. Other studies looked at the use of apps, these personality traits, and relational status. Thus, Timmermans and De Caluwé [71] found that single users of Tinder were more outgoing and open to new experiences than non-user singles, who scored higher in conscientiousness. For their part, Timmermans et al. [72] concluded that Tinder users who had a partner scored lower in agreeableness and conscientiousness and higher in neuroticism than people with partners who did not use Tinder.

The dark personality, on the other hand, has been used to predict the different reasons for using dating apps [48], as well as certain antisocial behaviors in Tinder [6,51]. As for the differences in dark personality traits between users and non-users of dating apps, the results are inconclusive. A study was localized that highlighted the relevance of psychopathy [3] whereas another study found no predictive power as a global indicator of dark personality [23].

### 3.3. Characteristics of Dating App Use

It is very difficult to know not only the actual number of users of dating apps in any country in the world but also the prevalence of use. This varies depending on the collectives studied and the sampling techniques used. Given this caveat, the results of some studies do allow an idea of the proportion of people using these apps. It has been found to vary between the 12.7% found by Castro et al. [23] and the 60% found by LeFebvre [44]. Most common, however, is to find a participant prevalence of between 40–50% [3,4,39,62,64], being slightly higher among men from sexual minorities [18,50].

The study of Botnen et al. [21] among Norwegian university students concluded that about half of the participants appeared to be a user of dating apps, past or present. But only one-fifth were current users, a result similar to those found by Castro et al. [23] among Spanish university students. The most widely used, and therefore the most examined, apps in the studies are Tinder and Grindr. The first is the most popular among heterosexuals, and the second among men of sexual minorities [3,18,36,70].

Findings from existing research on the characteristics of the use of dating apps can be divided among those referring to *before* (e.g., profiling), *during* (e.g., use), and *after* (e.g., offline behavior with other app users). Regarding *before*, the studies focus on users’ profile-building and self-presentation more among men of sexual minorities [52,77]. Ward [74] highlighted the importance of the process of choosing the profile picture in applications that are based on physical appearance. Like Ranzini and Lutz [59], Ward [74] mentions the differences between the “real self” and the “ideal self” created in dating apps, where one should try to maintain a balance between one and the other. Self-esteem plays a fundamental role in this process, as it has been shown that higher self-esteem encourages real self-presentation [59].

Most of the studies that analyze the use of dating apps focus on *during*, i.e. on how applications are used. As for the frequency of use and the connection time, Chin et al. [29] found that Tinder users opened the app up to 11 times a day, investing up to 90 minutes per day. Strubel and Petrie [67] found that 23% of Tinder users opened the app two to three times a day, and 14% did so once a day. Meanwhile, Sumter and Vandenbosch [3] concluded that 23% of the users opened Tinder daily.

It seems that the frequency and intensity of use, in addition to the way users behave on dating apps, vary depending on sexual orientation and sex. Members of sexual minorities, especially men, use these applications more times per day and for longer times [18]. As for sex, different patterns of behavior have been observed both in men and women, as the study of Timmermans and Courtois [4] shows. Men use apps more often and more intensely, but women use them more selectively and effectively. They accumulate more matches than men and do so much faster, allowing them to choose and have a greater sense of control. Therefore, it is concluded that the number of swipes and likes of app users does not guarantee a high number of matches in Tinder [4].

Some authors are alert to various behaviors observed in dating apps which, in some cases, may be negative for the user. For example, Yeo and Fung [77] mention the fast and hasty way of acting in apps, which is incongruous with cultural norms for the formation of friendships and committed relationships and ends up frustrating those who seek more lasting relationships. Parisi and Comunello [57] highlighted a key to the use of apps and a paradox. They referred to relational homophilia, that is, the tendency to be attracted to people similar to oneself. But, at the same time, this occurs in a context that increases the diversity of intimate interactions, thus expanding pre-existing networks. Finally, Licoppe [45] concluded that users of Grindr and Tinder present almost opposite types of communication and interaction. In Grindr, quick conversations seem to take precedence, aimed at organizing immediate sexual encounters, whereas, in Tinder, there are longer conversations and more exchange of information.

The latest group of studies focuses on offline behavior with contacts made through dating apps. Differences have been observed in the prevalence of encounters with other app users, possibly related to participants’ sociodemographic characteristics. Whereas Strugo and Muise [2], and Macapagal et al. [49] found that between 60 and 70% of their participants had had an encounter with another person known through these applications, in other studies this is less common, with prevalence being less than 50% [3,4,62]. In fact, Griffin et al. [39] stated that in-person encounters were relatively rare among users of dating apps.

There are also differences in the types of relationships that arose after offline encounters with other users. Strugo and Muise [2] concluded that 33% of participants had found a romantic partner and that 52% had had casual sex with at least one partner met through an app. Timmermans and Courtois [4] found that one-third of the offline encounters ended in casual sex and one-fourth in a committed relationship. Sumter and Vandenbosch [3], for their part, concluded that 18.6% of the participants had had sex with another person they had met on Tinder. And finally, the participants in the study of Timmermans and De Caluwé [71] indicated that: (1) they had met face-to-face with an average of 4.25 people whom they had met on Tinder; (2) they had had one romantic relationship with people met on Tinder; (3) they had had casual sex with an average of 1.57 people met on Tinder; and (4) they had become friends with an average of 2.19 people met on Tinder.

### 3.4. Motives for Dating App Use

There is a stereotype that dating apps are used only, or above all, to look for casual sex [44]. In fact, these applications have been accused of generating a hookup culture, associated with superficiality and sexual frivolity [2]. However, this is not the case. In the last five years, a large body of literature has been generated on the reasons why people use dating apps, especially Tinder, and the conclusion is unanimous: apps serve multiple purposes, among which casual sex is only one [1,4,44]. It has been found that up to 70% of the app users participating in a study [18] indicated that their goal when using it was not sex-seeking.

An evolution of research interest can be traced regarding the reasons that guide people to use dating apps [55]. The first classification of reasons for using Tinder was published by Ranzini and Lutz [59], who adapted a previous scale, designed for Grindr, composed of six motives: hooking up/sex (finding sexual partners), friendship (building a social network), relationship (finding a romantic partner), traveling (having dates in different places), self-validation (self-improvement), and entertainment (satisfying social curiosity). They found that the reason given by most users was those of entertainment, followed by those of self-validation and traveling, with the search for sex occupying fourth place in importance. However, the adaptation of this scale did not have adequate psychometric properties and it has not been reused.

Subsequently, Sumter et al. [68] generated a new classification of reasons to use Tinder, later refined by Sumter and Vandenbosch [3]. They proposed six reasons for use, both relational (love, casual sex), intrapersonal (ease of communication, self-worth validation), and entertainment (the thrill of excitement, trendiness). The motivation most indicated by the participants was that of love, and the authors concluded that Tinder is used: (1) to find love and/or sex; (2) because it is easy to communicate; (3) to feel better about oneself; and (4) because it’s fun and exciting.

At the same time, Timmermans and De Caluwé [70] developed the Tinder Motives Scale, which evaluates up to 13 reasons for using Tinder. The reasons, sorted by the scores obtained, were: to pass time/entertainment, curiosity, socializing, relationship-seeking, social approval, distraction, flirting/social skills, sexual orientation, peer pressure, traveling, sexual experience, ex, and belongingness. So far, the most recently published classification of reasons is that of Orosz et al. [55], who in the Tinder Use Motivations Scale proposed four groups of reasons: boredom (individual reasons to use Tinder to overcome boredom), self-esteem (use of Tinder to improve self-esteem), sex (use of Tinder to satisfy sexual need) and love (use of Tinder to find love). As in the previous scales, the reasons of seeking sex did not score higher on this scale, so it can be concluded that dating apps are not mainly used for this reason.

The existing literature indicates that reasons for the use of dating apps may vary depending on different sociodemographic and personality variables [1]. As for sex, Ranzini and Lutz [59] found that women used Tinder more for friendship and self-validation, whereas men used it more to seek sex and relationships. Sumter et al. [68] found something similar: men scored higher than women in casual sex motivation and also in the motives of ease of communication and thrill of excitement.

With regard to age, Ward [74] concluded that motivations change over time and Sumter et al. [68] found a direct association with the motives of love, casual sex, and ease of communication. In terms of sexual orientation, it has become commoner for people from sexual minorities, especially men, than for heterosexual participants to use these applications much more in the search for casual sex [18].

Finally, other studies have concluded that personality guides the motivations for the use of dating apps [3,72]. A line of research initiated in recent years links dark personality traits to the reasons for using Tinder. In this investigation, Lyons et al. [48] found that people who score high in Machiavellianism and psychopathy offer more reasons for use (e.g., get casual sex, acquiring social or flirting skills).

### 3.5. Benefits and Risks of Using Dating Apps

In the latter section, the benefits and advantages of the use of dating apps are analyzed. There is also an extensive literature on the risks associated with use. Many studies indicate that dating apps have opened a new horizon in how to meet potential partners, allowing access to many [3,6,8], which may be even more positive for certain individuals and groups who have been silenced or marginalized, such as some men from sexual minorities [80]. It has also been emphasized that these applications are a non-intimidating way to start connecting, they are flexible and free, and require less time and effort than other traditional means of communication [1,55].

On the other hand, the advantages of apps based on the technology they use and the possibilities they pose to users have been highlighted. Ranzini and Lutz [59] underlined four aspects. First is the portability of smartphones and tablets, which allows the use of apps in any location, both private and public. Second is availability, as their operation increases the spontaneity and frequency of use of the apps, and this, in turn, allows a quick face-to-face encounter, turning online interactions into offline relationships [70,77]. Thirdly is locatability, as dating apps allow matches, messages, and encounters with other users who are geographically close [77]. Finally is multimediality, the relevance of the visual, closely related to physical appearance, which results in two channels of communication (photos and messages) and the possibility of linking the profile with that of other social networks, such as Facebook and Instagram [4].

There is also considerable literature focused on the potential risks associated with using these applications. The topics covered in the studies can be grouped into four blocks, having in common the negative consequences that these apps can generate in users’ mental, relational, and sexual health. The first block focuses on the configuration and use of the applications themselves. Their emergence and popularization have been so rapid that apps pose risks associated with security, intimacy, and privacy [16,20]. This can lead to more insecure contacts, especially among women, and fears related to the ease of localization and the inclusion of personal data in apps [39]. Some authors highlight the paradox that many users suffer: they have more chances of contact than ever before, but at the same time this makes them more vulnerable [26,80].

This block can also include studies on the problematic use of apps, which can affect the daily lives of users [34,56], and research that focuses on the possible negative psychological effects of their use, as a link has been shown between using dating apps and loneliness, dissatisfaction with life, and feeling excluded from the world [24,34,78].

The second block of studies on the risks associated with dating apps refers to discrimination and aggression. Some authors, such as Conner [81] and Lauckner et al. [43], have argued that technology, instead of reducing certain abusive cultural practices associated with deception, discrimination, or abuse (e.g., about body types, weight, age, rural environments, racism, HIV stigma), has accentuated them, and this can affect users’ mental health. Moreover, certain antisocial behaviors in apps, such as trolling [6,51], have been studied, and a relationship has been found between being a user of these applications and suffering some episode of sexual victimization, both in childhood and adulthood [30].

The following block refers to the risks of dating app use regarding diet and body image. These applications, focusing on appearance and physical attractiveness, can promote excessive concerns about body image, as well as various negative consequences associated with it (e.g., unhealthy weight management behaviors, low satisfaction and high shame about the body, more comparisons with appearance [22,36,67,73]). These risks have been more closely associated with men than with women [61], perhaps because of the standards of physical attractiveness prevalent among men of sexual minorities, which have been the most studied collective.

The last block of studies on the risks of dating app use focuses on their relationship with risky sexual behaviors. This is probably the most studied topic in different populations (e.g., sexual minority men, heterosexual people). The use of these applications can contribute to a greater performance of risky sexual behaviors, which results in a higher prevalence of sexually transmitted illnesses (STIs). However, the results of the studies analyzed are inconclusive [40].

On the one hand, some studies find a relationship between being a user of dating apps and performing more risky sexual behaviors (e.g., having more sexual partners, less condom use, more relationships under the effects of alcohol and other drugs), both among men from sexual minorities [19] and among heterosexual individuals [32,41,62]. On the other hand, some research has found that, although app users perform more risky behaviors, especially having more partners, they also engage in more prevention behaviors (e.g., more sex counseling, more HIV tests, more treatment) and they do not use the condoms less than non-users [18,50,64,79]. Studies such as that of Luo et al. [46] and that of Wu [76] also found greater use of condoms among app users than among non-users.

Finally, some studies make relevant appraisals of this topic. For example, Green et al. [38] concluded that risky sexual behaviors are more likely to be performed when sex is performed with a person met through a dating app with whom some common connection was made (e.g., shared friends in Facebook or Instagram). This is because these users tend to avoid discussing issues related to prevention, either because they treat that person more familiarly, or for fear of possible gossip. Finally, Hahn et al. [40] found that, among men from sexual minorities, the contact time prior to meeting in person was associated with greater prevention. The less time between the conversation and the first encounter, the more likely the performance of risky behaviors.

## 4. Discussion

In a very few years, dating apps have revolutionized the way of meeting and interacting with potential partners. In parallel with the popularization of these applications, a large body of knowledge has been generated which, however, has not been collected in any systematic review. Given the social relevance that this phenomenon has reached, we performed this study to gather and analyze the main findings of empirical research on psychosocial content published in the last five years (2016–2020) on dating apps.

Seventy studies were located and analyzed, after applying stringent inclusion criteria that, for various reasons, left out a large number of investigations. Thus, it has been found that the literature on the subject is extensive and varied. Studies of different types and methodologies have been published, in very diverse contexts, on very varied populations and focusing on different aspects, some general and others very specific. Therefore, the first and main conclusion of this study is that the phenomenon of dating apps is transversal, and very present in the daily lives of millions of people around the world.

This transversality has been evident in the analysis of the characteristics of the users of dating apps. Apps have been found to be used, regardless of sex [59,68], age [49,58,71], sexual orientation [3,59], relational status [72], educational and income level [9,66], or personality traits [23,48,72].

Another conclusion that can be drawn from this analysis is that there are many preconceived ideas and stereotypes about dating apps, both at the research and social level, which are supported by the literature, but with nuances. For example, although the stereotype says that apps are mostly used by men, studies have concluded that women use them in a similar proportion, and more effectively [4]. The same goes for sexual orientation or relational status; the stereotype says that dating apps are mostly used by men of sexual minorities and singles [1], but some apps (e.g., Tinder) are used more by heterosexual people [3,59] and there is a remarkable proportion of people with a partner who use these apps [4,17].

A third conclusion of the review of the studies is that to know and be able to foresee the possible consequences of the use of dating apps, *how* and *why* they are used are particularly relevant. For this reason, both the use and the motives for use of these applications have been analyzed, confirming the enormous relevance of different psychosocial processes and variables (e.g., self-esteem, communication, and interaction processes), both *before* (profiling), *during* (use), and *after* (off-line encounters) of the use of dating apps.

However, in this section, what stands out most is the difficulty in estimating the prevalence of the use of dating apps. Very disparate prevalence have been found not only because of the possible differences between places and groups (see, for example [18,23,44,64]), but also because of the use of different sampling and information collection procedures, which in some cases, over-represent app users. All this hinders the characterization and assessment of the phenomenon of dating apps, as well as the work of the researchers. After selecting the group to be studied, it would be more appropriate to collect information from a representative sample, without conditioning or directing the study toward users, as this may inflate the prevalence rates.

The study of motives for the use of dating apps may contain the strongest findings of all those appraised in this review. Here, once again, a preconceived idea has been refuted, not only among researchers but across society. Since their appearance, there is a stereotype that dating apps are mostly used for casual sex [2,44]. However, studies constantly and consistently show that this is not the case. The classifications of the reasons analyzed for their use have concluded that people use dating apps for a variety of reasons, such as to entertain themselves, out of curiosity, to socialize, and to seek relationships, both sexual and romantic [3,59,68,70]. Thus, these apps should not be seen as merely for casual sex, but as much more [68].

Understanding the reasons for using dating apps provides a necessary starting point for research questions regarding the positive and negative effects of use [70]. Thus, the former result block reflected findings on the advantages and risks associated with using dating apps. In this topic, there may be a paradox in the sense that something that is an advantage (e.g., access to a multitude of potential partners, facilitates meeting people) turns into a drawback (e.g., loss of intimacy and privacy). Research on the benefits of using dating apps is relatively scarce, but it has stressed that these tools are making life and relationships easier for many people worldwide [6,80].

The literature on the risks associated with using dating apps is much broader, perhaps explaining the negative social vision of them that still exists nowadays. These risks have highlighted body image, aggression, and the performance of risky sexual behaviors. Apps represent a contemporary environment that, based on appearance and physical attractiveness, is associated with several negative pressures and perceptions about the body, which can have detrimental consequences for the physical and mental health of the individual [67]. As for assaults, there is a growing literature alerting us to the increasing amount of sexual harassment and abuse related to dating apps, especially in more vulnerable groups, such as women, or among people of sexual minorities (e.g., [12,82]).

Finally, there is considerable research that has analyzed the relationship between the use of dating apps and risky sexual behaviors, in different groups and with inconclusive results, as has already been shown [40,46,76]. In any case, as dating apps favor contact and interaction between potential partners, and given that a remarkable percentage of sexual contacts are unprotected [10,83], further research should be carried out on this topic.

### Limitations and Future Directions

The meteoric appearance and popularization of dating apps have generated high interest in researchers around the world in knowing how they work, the profile of users, and the psychosocial processes involved. However, due to the recency of the phenomenon, there are many gaps in the current literature on these applications. That is why, in general terms, more research is needed to improve the understanding of all the elements involved in the functioning of dating apps.

It is strange to note that many studies have been conducted focusing on very specific aspects related to apps while other central aspects, such as the profile of users, had not yet been consolidated. Thus, it is advisable to improve the understanding of the sociodemographic and personality characteristics of those who use dating apps, to assess possible differences with those who do not use them. Attention should also be paid to certain groups that have been poorly studied (e.g., women from sexual minorities), as research has routinely focused on men and heterosexual people.

Similarly, limitations in understanding the actual data of prevalence of use have been highlighted, due to the over-representation of the number of users of dating apps seen in some studies. Therefore, it would be appropriate to perform studies in which the app user would not be prioritized, to know the actual use of these tools among the population at large. Although further studies must continue to be carried out on the risks of using these applications (e.g., risky sexual behaviors), it is also important to highlight the positive sexual and relational consequences of their use, in order to try to mitigate the negative social vision that still exists about dating app users. Last but not least, as all the studies consulted and included in this systematic review were cross-sectional, longitudinal studies are necessary which can evaluate the evolution of dating apps, their users and their uses, motives, and consequences.

The main limitations of this systematic review concern the enormous amount of information currently existing on dating apps. Despite having applied rigorous exclusion criteria, limiting the studies to the 2016–2020 period, and that the final sample was of 70 studies, much information has been analyzed and a significant number of studies and findings that may be relevant were left out. In future, the theoretical reviews that are made will have to be more specific, focused on certain groups and/or problems.

Another limitation—in this case, methodological, to do with the characteristics of the topic analyzed and the studies included—is that not all the criteria of the PRISMA guidelines were followed [13,14]. We intended to make known the state of the art in a subject well-studied in recent years, and to gather the existing literature without statistical treatment of the data. Therefore, there are certain criteria of PRISMA (e.g., summary measures, planned methods of analysis, additional analysis, risk of bias within studies) that cannot be satisfied.

However, as stated in the Method section, the developers of the PRISMA guidelines themselves have stated that some systematic reviews are of a different nature and that not all of them can meet these criteria. Thus, their main recommendation, to present methods with adequate clarity and transparency to enable readers to critically judge the available evidence and replicate or update the research, has been followed [13].

Finally, as the initial search in the different databases was carried by only one of the authors, some bias could have been introduced. However, as previously noted, with any doubt about the inclusion of any study, the final decision was agreed between both authors, so we expect this possible bias to be small.

## 5. Conclusions

Dating apps have come to stay and constitute an unstoppable social phenomenon, as evidenced by the usage and published literature on the subject over the past five years. These apps have become a new way to meet and interact with potential partners, changing the rules of the game and romantic and sexual relationships for millions of people all over the world. Thus, it is important to understand them and integrate them into the relational and sexual life of users [76].

The findings of this systematic review have relevant implications for various groups (i.e., researchers, clinicians, health prevention professionals, users). Detailed information has been provided on the characteristics of users and the use of dating apps, the most common reasons for using them, and the benefits and risks associated with them. This can guide researchers to see what has been done and how it has been done and to design future research.

Second, there are implications for clinicians and health prevention and health professionals, concerning mental, relational, and sexual health. These individuals will have a starting point for designing more effective information and educational programs. These programs could harness the potential of the apps themselves and be integrated into them, as suggested by some authors [42,84].

Finally and unavoidably, knowledge about the phenomenon of dating apps collected in this systematic review can have positive implications for users, who may have at their disposal the necessary tools to make a healthy and responsible use of these applications, maximizing their advantages and reducing the risks posed by this new form of communication present in the daily life of so many people.

## Figures and Tables

**Table 1:** Characteristics of reviewed studies.

Author/s (Year)	Sample (*N*, Characteristics)	Methodology	Findings
Albury & Byron (2016) [16]	Same-sex attracted Australian men and women, aged between 18 and 29	Focus groups interviews	Mobile and apps contributed to participants’ perceptions of safety and risk when flirting or meeting with new sexual partners. Users strategically engaged with the security features of apps to block unwanted approaches and to manage privacy concerns when interacting with others.
Alexopoulos et al. (2020) [17]	395 participants, recruited through a U.S.-based university and Amazon Mechanical Turk, both sexes (*M =* 26.7, *SD* = 8.32)	Online survey	People´s perceived success on a dating app was positively associated with their intention to commit infidelity through perceived amount of available partners.
Badal et al. (2018) [18]	3105 males identified as gay or bisexual, aged 18–64 (*M* = 32.35, *SD* = 9.58), residents in the United States or Puerto Rico	Web-based survey	More than half (55.7%) of participants were frequent users of dating websites and apps. Two third (66.7%) of users had casual partner only in the prior 12 months and reported a high average number of casual sex partners in the previous 12 months compared to never users. The most frequently used dating apps was Grindr (60.2%).
Boonchutima & Kongchan (2017) [19]	350 Thai men who have sex with men	Online survey	73% of participants were dating app users, to find potential partners as well as for inviting others into illicit drug practice. Persuasion through dating apps influenced people toward accepting the substance use invitation, with a 77% invitation success rate. Substance use was linked with unprotected sex.
Boonchutima et al. (2016) [20]	286 gay dating app users in Thailand	Online survey	There are positive associations between the degree of app usage and the amount of information being disclosed. Moreover, the frequency of usage and the disclosure of personal information were associated with a higher rate of unprotected sex.
Botnen et al. (2018) [21]	641 Norwegian university students, both sexes, aged between 19 and 29 (*M* = 21.4, *SD* = 1.6)	Offline questionnaire	Nearly half of the participants reported former or current dating app use. 20% was current users. Dating app users tend to report being less restricted in their sociosexuality than participants who have never used apps. This effect was equally strong for men and women.
Breslow et al. (2020) [22]	230 sexual minority men, U.S.-located	Online survey	The number of apps used was positively related with objectification, internalization, and body surveillance, and negatively related with body satisfaction and self-esteem.
Castro et al. (2020) [23]	1705 students from a Spanish university, both sexes, aged between 18 and 26 (*M* = 20.60, *SD* = 2.09)	Online survey	Men, older youths, members of sexual minorities, and people without partner were more likely to be dating app users. In addition, some traits of the Big Five (openness to experience) allowed prediction of the current use of dating apps. The dark personality showed no predictive ability.
Chan (2017) [24]	401 men who have sex with men, U.S.-located, ages ranged from 18 to 44 years (*M* = 23.45, *SD* = 4.09)	Online survey	There was a significant relationship between sex-seeking and the number of casual sex partners, mediated by the intensity of apps use. Furthermore, gay identity confusion and outness to the world moderated these indirect effects.
Chan (2017) [25]	257 U.S. citizens, both sexes, aged between 18 and 34 (*M* = 27.1, *SD* = 4.35), heterosexuals.	Online survey (via Qualtrics)	Regarding using dating apps to seek romance, people´s attitude and perceived norms were predictive of such intent. Sensation-seeking and smartphone use had a direct relationship with intent. Regarding using dating apps for seeking sex, people´s attitude and self-efficacy were predictive of such intent.
Chan (2018) [26]	(1) 7 Asian-American users of gay male dating apps, aged between 26 and 30; (2) 245 U.S. male dating app users, aged between 19 and 68.	(1) semi-structured interviews; (2) online survey	Users reported ambivalence in establishing relationships, which brought forth the ambiguity of relationships, dominance of profiles, and over-abundance of connections on these apps.
Chan (2018) [27]	19 female dating app users in China, aged between 21 and 38	Semi-structured interviews	Female dating app users offered multiple interpretations of why they use dating apps (e.g., sexual experience, looking for a relationship, entertainment). They also face several challenges in using dating apps (e.g., resisting social stigma, assessing men´s purposes, undesirable sexual solicitations).
Chan (2019) [28]	125 male heterosexual active users of dating apps (Momo) in urban cities in China, aged between 18 and 47 (*M* = 28.94, *SD* = 5.96)	Online survey (via Qualtrics)	The endorsement of masculinity had an indirect positive relationship with the number of sex partners mediated by the sex motive. At the same time, this had a direct but negative association with the number of sex partners. These paradoxical associations were explained by different patterns across the individual dimension of masculinity ideology (e.g., importance of sex, avoidance of femininity).
Chin et al. (2019) [29]	183 North-American adults, both sexes, aged between 18 and 65 (*M* = 29.97, *SD* = 8,50). Recruited via Amazon´s Mechanical Turk.	Online survey	People with a more anxious attachment orientation were more likely to report using dating apps than people lower in anxiety attachment. People with a more avoidance attachment orientation were less like to report using dating apps than people lower in avoidant attachment. The most common reason people reported for using apps was to meet others, and the most common reason people reported for not using apps was difficulty trusting people online.
Choi et al. (2016) [30]	666 university students from Hong Kong, both sexes (*M* = 20.03, *SD* = 1.52)	Self-administered survey (not online)	Users of dating apps were more likely to have unprotected sex with a casual sex partner the last time they engaged in sexual intercourse. Using dating apps for more than 12 months was associated with having a casual sex partner in the last episode of sexual intercourse, as well as having unprotected sex with that casual partner.
Choi et al. (2016) [31]	666 university students from Hong Kong, both sexes (*M* = 20.03, *SD* = 1.52)	Self-administered survey (not online)	Users of dating apps and current drinkers were less likely to have consistent condom use. Users of dating apps, bisexual/homosexual subjects, and female subjects were more likely not to have used condoms the last time they had sexual intercourse.
Choi et al. (2017) [32]	666 university students from Hong Kong, both sexes (*M* = 20.03, *SD* = 1.52)	Self-administered survey (not online)	The use of dating apps for more than one year was found to be associated with recreational drug use in conjunction with sexual activities. Other risk factors of recreational drug use in conjunction with sexual activities included being bisexual/homosexual, male, a smoker, and having one´s first sexual intercourse before 16 years. The use of dating apps was not a risk factor for alcohol consumption in conjunction with social activities.
Choi et al. (2017) [33]	666 university students from Hong Kong, both sexes (*M* = 20.03, *SD* = 1.52)	Self-administered survey (not online)	Users of dating apps were more likely to have been sexually abused in the previous year than non-users. Using dating apps was also a risk factor for lifetime sexual abuse.
Coduto et al. (2020) [34]	269 undergraduate students, both sexes, aged between 18 and 24 (*M* = 20.85, *SD* = 2.45)	Online survey	The data provided support for moderated serial mediation. This type of mediation predicted by the social skills model was significant only among those high in loneliness, with positive association between preference for online social interaction and compulsive use being significant among those with high in loneliness.
Duncan & March (2019) [6]	587 Tinder users, both sexes (*M* = 23.75, *SD* = 6.05)	Online survey	They created and validated the Antisocial Uses of Tinder Scale. Exploratory and confirmatory factor analyses revealed three forms of antisocial behavior (general, esteem, and sexual). Regression analyses showed the predictive utility of gender and the dark traits across antisocial behaviors.
Ferris & Duguay (2020) [35]	27 women seeking women (WSW) from Australia, Canada, and the UK, aged between 19 and 35.	Semi-structured interviews	Participants perceived that they were entering a space conducive to finding women seeking women. However, men, couples, and heterosexual women permeated this space, heightening the need for participants to signal non-heterosexual identity.
Filice et al. (2019) [36]	13 men who have sex with men, aged between 18 and 65 (*M* = 29).	Semi-structured interviews	Grindr affects user body image through three primary mechanisms: weight stigma, sexual objectification and social comparison. Moreover, participants identified several protective factors and coping strategies.
Gatter & Hodkinson (2016) [8]	75 participants, both sexes, aged between 20 and 69, divided in three groups (Tinder users, online dating agency users, and non-users).	Online survey	No differences were found in motivations, suggesting that people may use both online dating agencies and Tinder for similar reasons. Tinder users were younger than online dating agency users, which accounted for observed group differences in sexual permissiveness. There were no differences in self-esteem or sociability between the groups. Men were more likely than women to use both types of dating and scored higher in sexual permissiveness.
Goedel et al. (2017) [37]	92 men who have sex with men, Grindr users, aged between 18 and 70.	Online survey	Obese participants scored significantly higher on measures of body dissatisfaction and lower on measures of sexual sensation seeking. Decreased propensities to seek sexual-sensation were associated with fewer sexual partners.
Green et al. (2018) [38]	953 university students, both sexes, aged between 18 and 24 (*M* = 20.76, *SD* = 1.81)	Online survey	Tinder users may: (1) perceive partners with whom they share “common connections” as familiar or “safe”, which may give users a false sense of security about the sexual health risks; or (2) be hesitant to discuss sexual health matters with partners who are within their sexual network due to fear of potential gossip. Both lines of thought may reduce safer sex behaviors.
Griffin et al. (2018) [39]	409 U.S. university students, heterosexuals, both sexes (*M* = 19.7, *SD* = 7.2)	Online survey	39% of participants had used a dating app, and 60% of them were regular users. Tinder was the most popular dating app. Top reasons for app use were fun and to meet people. Very few users (4%) reported using apps for casual sex encounters, although many users (72% of men and 22% of women) were open to meeting a sexual partner with a dating app. Top concerns included safety and privacy.
Hahn et al. (2018) [40]	Study 1: 64 men who have sex with men dating app users, aged between 18 and 24 (*M* = 22.66, *SD* = 1.38). Study 2: 217 participants, both sexes, aged between 18 and 21 (*M* = 20.23, *SD* = 0.85). Recruited by Amazon Mechanical Turk (both studies).	Online survey (both studies)	Study 1: those who talked less before meeting in person engaged in more sexual risk behaviors than those who spent more time talking before meeting in person. Study 2: there were no differences in sexual risk behaviors between dating app users and non-users. However, when examining app users by time before meeting, those with a shorter time before meeting were more impulsive and more likely to report sexual risk behaviors.
Hart et al. (2016) [41]	539 heterosexual attenders of two genito-urinary medicine clinics, both sexes (*Mdn* = 21–30 years).	Self-administered survey	A quarter of participants use apps to find partners online. This study identified high rates of sexually transmitted infections, condomless use and recreational drug use among app users.
Kesten et al. (2019) [42]	25 men who have sex with men residents in England aged between 26 and 57 years (*Mdn* = 30–39).	Semi-structured interviews	Sexual health information delivery through social media and dating apps was considered acceptable. Concerns were expressed that sharing or commenting on social media sexual health information may lead to judgments and discrimination. Dating apps can easily target men who have sex with men.
Lauckner et al. (2019) [43]	20 sexual minority males living in U.S. non-metropolitan areas, aged between 18 and 60.	Survey and semi-structured interviews	Many participants reported negative experiences while using dating apps. Specifically, they discussed instances of deception or “catfishing”, discrimination, racism, harassment, and sexual coercion.
LeFebvre (2018) [44]	395 participants recruited from Amazon Mechanical Turk, both sexes, aged between 18 and 34 (*M* = 26.41, *SD* = 4.17)	Online survey	The prevalent view that Tinder is a sex or hookup app remains salient among users; although, many users utilize Tinder for creating other interpersonal communication connections and relationships, both romantic and platonic. Initially, Tinder users gather information to identify their preferences.
Licoppe (2020) [45]	Grindr study: 23 male users of Grindr in Paris. Tinder study: 40 male and female users of Tinder in France.	In-depth interviews	Grindr and Tinder users take almost opposite conversational stances regarding the organization of casual hookups as sexual, one-off encounters with strangers. While many gay Grindr users have to chat to organize quick sexual connections, many heterosexual Tinder users are looking to achieve topically-rich chat conversations.
Luo et al. (2019) [46]	9280 men who have sex with men dating app users in China (*Mdn* = 31–40 years).	Online survey	Results indicated that frequent app use was associated with lower odds of condomless anal intercourse among men who have sex with men in China.
Lutz & Ranzini (2017) [47]	497 U.S.-based participants, both sexes (*M* = 30.9, *SD* = 8.2), recruited through Amazon Mechanical Turk.	Online survey	Tinder users were more concerned about institutional privacy than social privacy. Moreover, different motivations for using Tinder (hooking up, relationship, friendship, travel, self-validation, entertainment) affect social privacy concerns more strongly than institutional concerns. Finally, loneliness significantly increases users´ social and institutional privacy concerns.
Lyons et al. (2020) [48]	216 current or former Tinder users, from UK, USA and Canada, both sexes, aged between 18 and 56 (*M* = 22.87, *SD* = 7.09).	Online survey	Using Tinder for acquiring sexual experience was related to being male and being high in psychopathy. Psychopathy was positively correlated with using Tinder to distract oneself from other tasks. Higher Machiavellianism and being female were related to peer pressure as a Tinder use motivation. Using Tinder for acquiring social or flirting skills had a negative relationship with narcissism, and a positive relationship with Machiavellianism. Finally, Machiavellianism was also a significant, positive predictor of Tinder use for social approval and to pass the time.
Macapagal et al. (2019) [49]	219 adolescent members of sexual and gender minorities assigned male at birth, U.S.-located, aged between 15 and 17 (*M* = 16.30, *SD* = 0.74).	Online survey	Most participants (70.3%) used apps for sexual minority men, 14.6% used social media/other apps to meet partners, and 15.1% used neither. Nearly 60% of adolescents who used any type of app reported having met people from the apps in person. Dating apps and social media users were more like to report condomless receptive anal sex.
Macapagal et al. (2018) [50]	200 adolescent men who have sex with men, aged between 14 and 17 (*M* = 16.64, *SD* = 0.86).	Online survey	52.5% of participants reported using gay-specific apps to meet partner for sex. Of these, most participants reported having oral (75.7%) and anal sex (62.1%) with those partners. Of those who reported having anal sex, only 25% always used condoms.
March et al. (2017) [51]	357 Australian adults, both sexes, aged between 18 and 60 (*M* = 22.50, *SD* = 6.55).	Online survey	Traits of psychopathy, sadism, and dysfunctional impulsivity were significantly associated with trolling behaviors. Subsequent moderation analyses revealed that dysfunctional impulsivity predicts perpetration of trolling, but only if the individual has medium or high levels of psychopathy.
Miller (2019) [52]	322 North-American men who have sex with men apps users, aged between 18 and 71 (*M* = 30.6).	Online survey	Results indicated that the majority of men presented their face in their profile photo and that nearly one in five presented their unclothed torso. Face-disclosure was connected to higher levels of app usage, longer-term app usage, and levels of outness. The use of shirtless photos was related to age, a higher drive for muscularity, and more self-perceived masculinity.
Miller & Behm-Morawitz (2016) [53]	143 men who have sex with men app users, aged between 18 and 50 (*M* = 27.41, *SD* = 7.60).	Online experiment	Results indicated that the use of femmephobic language in dating profiles affects a potential partner´s perceived intelligence, sexual confidence, and dateability, as well as one´s desire to meet potential partners offline for friendship or romantic purposes.
Numer et al. (2019) [54]	16 gay/bisexual Canada-located males, Grindr users, aged between 20 and 50.	Semi-structured interviews	Three threads of disclosure emerged: language and images, filtering, and trust. These threads of disclosure provide insights into how the sexual beliefs, values, and practices of gay and bisexual men who have sex with men are shaped on dating apps.
Orosz et al. (2018) [55]	Study 1: 414 participants, both sexes, aged between 18 and 43 (*M* = 22.71, *SD* = 3.56). Study 2: 346 participants, both sexes, aged between 18 and 51 (*M* = 22.02, *SD* = 3.41). Study 3: 298 participants, both sexes, aged between 19 and 65 (*M* = 25.09, *SD* = 5.82)	Online survey (via Qualtrics)	Study 1: a 16-item first-order factor structure was identified with four motivational factors (sex, love, self-esteem enhancement, boredom). Study 2: problematic Tinder use was mainly related to using Tinder for self-esteem enhancement. The Big Five personality factors were only weakly related to the four motivations and to problematic Tinder use. Study 3: showed that instead of global self-esteem, relatedness-need frustration was the strongest predictor of self-esteem enhancement Tinder use motivation that, in turn, was the strongest predictor of problematic Tinder use.
Orosz et al. (2016) [56]	430 Hungarian participants, both sexes, aged between 18 and 51 (*M* = 22.53, *SD* = 3.74).	Online survey	They created and validated the Problematic Tinder Use Scale (PTUS). Both the 12- and the 6-item versions were tested. The 6-item unidimensional structure has appropriate reliability and factor structure. No salient demographic-related differences were found.
Parisi & Comunello (2020) [57]	20 Italian dating app users, both sexes, aged between 22 and 65 (*M* = 38).	Focus groups	Participants appreciated the role of mobile dating apps in reinforcing their relational homophile (their tendency to like people that are “similar” to them) whilst, at the same time, mainly using these apps for increasing the diversity of their intimate interactions in terms of extending their networks.
Queiroz et al. (2019) [58]	412 men who have sex with men dating app users, located in Brazil, with ages over 50 years.	Online survey	Factors associated with a higher chance of having HIV were: sexual relations with an HIV-infected partner, chemsex and, above all, having an HIV-infected partner. The belief that apps increase protection against STI, and not being familiar with post-exposure prophylaxis, were associated with decreased chances of having HIV.
Ranzini & Lutz (2017) [59]	497 U.S.-based participants, both sexes (*M* = 30.9, *SD* = 8.2), recruited through Amazon Mechanical Turk	Online survey (via Qualtrics)	Self-esteem was the most important psychological predictor, fostering real self-presentation but decreasing deceptive self-presentation. The motives of use (hooking up/sex, friendship, relationship, traveling, self-validation, entertainment) also affect self-presentation, and were related to demographic characteristics and psychological antecedents.
Rochat et al. (2019) [60]	1159 heterosexual Tinder users, both sexes, aged between 18 and 74 (*M* = 30.02, *SD* = 9.19).	Online survey	Four reliable clusters were identified: two with low levels of problematic use (“regulated” and “regulated with low sexual desire”), one with and intermediate level of problematic use (“unregulated-avoidant”), and one with a high-level of problematic use (“unregulated-avoidant”). The clusters differed on gender, marital status, depressive mood, and use patterns.
Rodgers et al. (2019) [61]	170 college students, both sexes, aged between 18 and 32 (*M* = 22.2)	Online survey	Among males, frequent checking of dating apps was positively correlated with body shame and negatively with beliefs regarding weight/shape controllability. Media internalization was negatively correlated with experiencing negative feelings when using dating apps, and positively with positive feelings. Few associations emerged among females.
Sawyer et al. (2018) [62]	509 students from an U.S. university, both sexes, aged between 18 and 25 (*M* = 20.07, *SD* = 1.37).	Online survey	39.5% of the participants reported using dating apps. Individuals who used dating apps had higher rates of sexual risk behavior in the last three months, including sex after using drugs or alcohol, unprotected sex (anal or vaginal), and more lifetime sexual partners.
Schreus et al. (2020) [63]	286 participants, both sexes, aged between 18 and 30 (*M* = 24.60, *SD* = 3.41).	Online survey (via Qualtrics)	More frequent dating app use was positively related to norms and beliefs about peers´ sexting behaviors with unknown dating app matches (descriptive norms), norms beliefs about peers´ approval of sexting with matches (subjective norms), and negatively related to perceptions of danger sexting with matches (risk attitudes).
Sevi et al. (2018) [7]	163 U.S.-located Tinder users, both sexes, aged between 18 and 53 (*M* = 27.9, *SD* = 6.5), recruited through Amazon Mechanical Turk.	Online survey	Sexual disgust sensitivity and sociosexuality were predictors of motivation to use Tinder for casual sex. The participants with higher sexual disgust sensitivity reported a lower motivation while the participants with higher sociosexuality reported a higher motivation for casual sex in their Tinder usage. While this model explained the motivation for men, a different model explained women´s motivation. Sociosexuality mediated the relationship between sexual disgust sensitivity and the motivation to use Tinder for casual sex for women Tinder users.
Shapiro et al. (2017) [64]	415 students from a Canadian university, both sexes, aged between 18 and 26 (*M* = 20.73, *SD* = 1.73).	Online survey (via Qualtrics)	Greater likelihood of using Tinder was associated with a higher level of education and greater reported need for sex, while decreased likelihood of using Tinder was associated with a higher level of academic achievement, lower sexual permissiveness, living with parents or relatives, and being in a serious relationship. Higher odds of reporting nonconsexual sex and having five or more previous sexual partners users were found in Tinder users. Tinder use was not associated with condom use.
Solis & Wong (2019) [65]	433 Chinese dating app users, both sexes, aged between 11 and 50 (*M* = 30).	Online survey	Sexuality was the only predictor of the reasons that people use dating apps to meet people offline for dates and casual sex. Among the perceived risks of mobile dating, only the fear of self-exposure to friends, professional networks, and the community significantly explained why users would not meet people offline for casual sex.
Srivastava et al. (2019) [66]	253 homeless youth located in Los Angeles, both sexes, aged between 14 and 24 (*M* = 21.9, *SD* = 2.16).	Computer-administered survey	Sexual minority (43.6%) and gender minority (12.1%) youth reported elevated rates of exchange sex compared to cisgender heterosexual youth. 23% of youth who engaged in survival or exchange sex used dating apps or websites to find partners. Exchange sex and survival sex were associated with having recent HIV-positive sex partners.
Strubel & Petrie (2017) [67]	1,147 U.S.-located single participants, both sexes, aged between 18 and 34.	Online survey	Tinder users, regardless of gender, reported significantly lower levels of satisfaction with face and body and higher levels of internalization, appearance comparisons, and body shame and surveillance than non-users. For self-esteem, male Tinder users scored significantly lower than the other groups.
Strugo & Muise (2019) [2]	Study 1: 334 Tinder users, both sexes. Study 2: 441 single Tinder users, both sexes, aged between 18 and 59 (*M* = 27.7, *SD* = 6.6), recruited via Amazon Mechanical Turk.	Online survey	Study 1: higher approach goals for using Tinder, such as to develop intimate relationships, were associated with more positive beliefs about people on Tinder, and, in turn, associated with reporting greater perceived dating success. In contrast, people with higher avoidance goals, reported feeling more anxious when using Tinder. Study 2: previous results were not accounted for by attractiveness of the user and were consistent between men and women, but differed based on the age of user.
Sumter & Vandenbosch (2019) [3]	541 participants, both sexes, aged between 18 and 30 (*M* = 23.71, *SD* = 3.29).	Online survey (via Qualtrics)	Nearly half of the sample used dating apps regularly, with Tinder being the most popular. Non-users were more likely to be heterosexual, high in dating anxiety, and low in sexual permissiveness than dating app users. Among app users, dating app motivations (relational, interpersonal, entertainment), were meaningfully related to identity features.
Sumter et al. (2017) [68]	266 Dutch young, both sexes, aged between 18 and 30 (*M* = 23.74, *SD* = 2.56).	Online survey (via Qualtrics)	They found six motivations to use Tinder (love, casual sex, ease of communication, self-worth validation, thrill of excitement, trendiness). The Love motivation appeared to be a stronger motivation to use Tinder than the Casual sex motivation. Men were more likely to report a Casual sex motivation for using Tinder than women. With regard to age, the motivations Love, Casual Sex, and Ease of communication were positively related to age.
Tang (2017) [69]	12 Chinese lesbian and bisexual women, aged 35 and above.	In-depth interviews	Although social media presents ample opportunities for love and intimacy, the prevailing conservative values and cultural norms surrounding dating and relationships in Hong Kong are often reinforced and played out in their choice of romantic engagement.
Timmermans & Courtois (2018) [4]	1038 Belgian Tinder users, both sexes, aged between 18 and 29 (*M* = 21.80, *SD* = 2.35).	Online survey	User´s swiping quantity does not guarantee a higher number of Tinder matches. Women have generally more matches than men and men usually have to start a conversation on Tinder. Less than half of the participants reported having had an offline meeting with another Tinder user. More than one third of these offline encounters led to casual sex, and more than a quarter resulted in a committed relationship.
Timmermans & De Caluwé (2017) [70]	Study 1: 18 students from an U.S. university, between 18 and 24 years. Study 2: 1728 Belgian Tinder users, both sexes, aged between 18 and 67 (*M* = 22.66, *SD* = 4.28). Study 3: 485 Belgian Tinder users, both sexes, aged between 19 and 49 (*M* = 26.71, *SD* = 5.32). Study 4: 1031 Belgian Tinder users, both sexes, aged between 18 and 69 (*M* = 26.93, *SD* = 7.93).	Study 1: semi-structured interviews. Studies 2–4: online survey	The Tinder Motives Scale (TMS) consists of 58 items and showed a replicable factor structure with 13 reliable motives (social approval, relationship seeking, sexual experience, flirting/social skills, travelling, ex, belongingness, peer pressure, socializing, sexual orientation, pass time/entertainment, distraction, curiosity). The TMS is a valid and reliable scale to assess Tinder use motivations.
Timmermans & De Caluwé (2017) [71]	502 single Belgian participants, both sexes, aged between 18 and 29 (*M* = 23.11, *SD* = 2.83).	Online survey	Single Tinder users were more extraverted and open to new experiences than single non-users, whereas single non-users tended to be more conscientious than single users. Additionally, the findings provide insights into how individual differences (sociodemographic and personality variables) in singles can account for Tinder motives.
Timmermans et al. (2018) [72]	Sample 1: 1616 participants, both sexes, aged between 18 and 74 (*M* = 28.90, *SD* = 10.32). Sample 2: 1795 participants, both sexes, aged between 18 and 58 (*M* = 22.89, *SD* = 4.57).	Online survey	Non-single Tinder users differed significantly on nine Tinder motives from single Tinder users. Non-single users generally reported a higher number of romantic relationships and casual sex relationships with other Tinder users compared to single Tinder users. Non-single Tinder users scored significantly lower on agreeableness and conscientiousness, and significantly higher on neuroticism and psychopathy compared to non-users in a committed relationship.
Tran et al. (2019) [73]	1726 U.S.-located participants, both sexes, aged between 18 and 65, recruited through Amazon Mechanical Turk	Online survey	Dating app users had substantially elevated odds of unhealthy weight control behaviors compared with non-users. These findings were supported by results of additional gender-stratified multivariate logistic regression analyses among women and men.
Ward (2017) [74]	21 Dutch participants, recruited in Tinder, both sexes, aged between 19 and 52 years.	Semi-structured interviews	Users´ motivations for using Tinder ranged from entertainment to ego-boost to relationship seeking, and these motivations sometimes change over time. Profile photos are selected in an attempt to present an ideal yet authentic self. Tinder users “swipe” not only in search of people they like, but also for clues as to how to present themselves in order to attract others like them.
Weiser et al. (2018) [75]	550 students from an U.S.- university, both sexes, aged between 18 and 33 (*M* = 20.86, *SD* = 1.82).	Online survey	Participants indicated that most knew somebody who had used Tinder to meet extradyadic partners, and several participants reported that their own infidelity had been facilitated by Tinder. Sociosexuality and intentions to engage in infidelity were associated with having used Tinder to engage in infidelity.
Wu (2019) [76]	262 participants, both sexes, aged between 18 and 30 (*M* = 23.14, *SD* = 2.11).	Online survey	Tinder users reported higher scores for sexual sensation seeking and sexual compulsivity than non-users. No differences were found regarding risky sexual behavior, except that Tinder users use condoms more frequently than non-users.
Wu & Ward (2019) [11]	21 Chinese urban dating app users, aged between 20 and 31 (*M* = 25.3).	Semi-structured interviews	Casual sex is perceived as a form of social connection with the potential to foster a relationship.
Yeo & Fung (2018) [77]	74 gay mobile dating app users, aged between 18 and 26 years	Semi-structured interviews and focus groups	The accelerated tempo of interactions facilitated by perpetual connectivity, mutual proximity awareness, and instant messaging was seen to entail instantaneous and ephemeral relationships. The interface design, which foregrounds profile photos and backgrounds textual self-descriptions, was perceived to structure the sequence of browsing and screening in favor of physical appearance and users seeking casual hook-ups.
Zervoulis et al. (2019) [78]	191 men who have sex with men living in the United Kingdom aged between 18 and 72 (*M* = 36.51, *SD* = 10.17).	Online survey	High users of dating apps reported a lower sense of community, higher levels of loneliness, and lower levels of satisfaction with life. There was some evidence that those men who have sex with men who use dating apps mainly for sexual encounters reported higher levels of self-esteem and of satisfaction with life compared to those who used dating apps mainly for other reasons.
